# Association of hypertension with risk factors & hypertension related behaviour among the aboriginal Nicobarese tribe living in Car Nicobar Island, India

**Published:** 2011-03

**Authors:** Sathya Prakash Manimunda, Attayuru Purushottaman Sugunan, Vivek Benegal, Nagalla Balakrishna, Mendu Vishnuvardhana Rao, Kasturi S. Pesala

**Affiliations:** * *Regional Medical Research Centre (ICMR), Port Blair, India*; + *National Institute of Mental Health & Neuro Sciences, Bangalore, India*; # *National Institute of Nutrition (ICMR), Hyderabad, India*; ## *Indira Gandhi National Open University, Port Blair, India*

**Keywords:** Aboriginal, alcohol, Andaman and Nicobar islands, behaviour, hypertension, India, indigenous, Nicobarese, obesity, overweight, tobacco

## Abstract

**Background & objectives:**

There are no composite estimates on prevalence of hypertension among indigenous tribes in India. The present study was carried out to estimate the prevalence of hypertension, its association with determinants, and to assess the hypertension related behaviour of the Nicobarese aborigines.

**Methods:**

This cross-sectional survey was carried during 2007 and 2009. Subjects were chosen by two stage design. Total 975 subjects of 1270 (response rate of 76.8%) were investigated (M: 43.5%; F: 56.5%). The data were collected by history, clinical examination (blood pressure), and examination (weight and height). The association of hypertension with age, education, tobacco, alcohol consumption and their dependency status (KF score, QF value, and AUDIT score) and nutritional status was estimated by bivariate regression analysis; the increasing trend in the prevalence of hypertension with increasing age and decreasing educational status was analysed by χ^2^ for linear trend. Significant variables in bivariate regression analysis (age, education, alcohol consumption status, and nutritional status) were subjected to multiple logistic regression analysis (MLR).

**Results:**

The prevalence of hypertension was 50.5 per cent [95% CI 46.1-54.9 (M: 50.7%; F: 50.3%)]. The prevalence of tobacco, alcohol consumption, and overweight/obesity was 88, 54, and 37 per cent respectively. The bivariate analysis has shown association between hypertension and age, education subcategories, alcohol consumption, and overweight/obesity (*P*<0.05). The increasing trend in the prevalence of hypertension with increasing age (χ^2^ for linear trend=95.88, *P*< 0.001) and decreasing educational status (χ^2^ for linear trend=25.55, *P*< 0.001) was statistically significant. MLR analysis revealed a significant association between hypertension and various age categories and overweight/obesity.

**Interpretation & conclusions:**

The findings of the present study highlight high prevalence of hypertension among Nicobarese aborigenes.

Hypertension is one of the important public health challenges worldwide because of its high frequency and concomitant risks of cardiovascular and kidney disease^1^,^2^. It has been identified as a leading risk factor for mortality and ranked third as a cause of disability-adjusted life-years^3^. The accelerating epidemic of hypertension in India was documented by studies done at various places across the country^4^. The National Nutrition Monitoring Bureau (NNMB), which monitors the nutritional status of the population in nine States of India has estimated the prevalence of hypertension among the rural adult (aged 18 and above) population of India to be 25 per cent during 2004-2005^5^.

Studies carried out worldwide, on indigenous tribes who are on the process of acculturation whether it may be American Indians, tribes of Malaysia, South America or Africa, has shown the prevalence of hypertension in the range of 10-35 per cent^6^–^9^. In India, there is no composite estimate on prevalence of hypertension among indigenous tribes, but the increasing prevalence of hypertension across the time among tribes has been observed by independent researchers. Isolated studies carried out in these populations like among Lepchas of Sikkim Himalayas, tribes of Andhra Pradesh, Gujarat, and Orissa have documented the hypertension prevalence in the range of 15 to 42 per cent^10^–^13^.

The Andaman and Nicobar Islands is an archipelago, situated in the Bay of Bengal, are home to six indigenous population groups. The indigenous tribes account for about 8 per cent (30000 of 350000) of the islands’ population^14^. The Nicobarese constitute more than 95 per cent (29000 of 30000) of these populations. The Nicobar district is situated south of Andaman group of islands. The northern-most island of the Nicobar group, Car Nicobar Island, has 15 villages; with a population of 20292 Nicobarese tribes people. Largely homogenous in terms of genetic make-up, occupation, and income status, around 75 per cent of the population is literate. Living in extended- joint families or “tuhets”, they eke out their livelihood by cultivating coconut, areca-nut, banana, and pig rearing^15^,^16^. Consumption of smokeless tobacco in the form of chewing and alcoholic beverage, “toddy” derived from the efflorescent spathe of the coconut palm is widely prevalent among the Nicobarese tribe. The process of acculturation and consequent nutritional transition is evident among Nicobarese in recent times. The present study was planned, with the objective of estimating the prevalence of hypertension, the association, and risk estimates of established risk factors of hypertension, and the awareness levels and compliance with medication advice, within the Nicobarese tribes people living in Car Nicobar Island.

## Material & Methods

The study protocol was approved by the Institutional Ethics Committee of Regional Medical Research Centre, Port Blair.

### 

#### Study design and period:

This cross-sectional study was carried out from September 2007 to November 2007. A repeat survey was carried out in March 2009 to April 2009 to assess the compliance with medication advice.

#### Sampling strategy and sample size:

A 2-stage sampling design was employed. In the first stage, 40 of 308 “tuhets” (extended joint families) of the island (from a total of 15 villages) were randomly selected, and in the second stage, all subjects aged 18 yr or more in each selected tuhet were investigated. As there is no estimate of hypertension among the Nicobarese community, the prevalence of hypertension *i.e*., 25 per cent estimated by NNMB^5^ among the rural adult population of main land India was taken as reference for arriving at the projected sample size. Assuming a prevalence of 25 per cent and absolute precision of 5 per cent with 95 per cent confidence, the required sample size was estimated to be 288. To compensate for increased sampling error due the deployment of a 2-stage design instead of simple random sampling and losses in coverage, the target was thereafter raised to 1000. Total 975 of 1270 eligible (response rate of 76.8%) Nicobarese aged 18 and above were investigated.

For the repeat survey, a subpopulation (250) of the previously diagnosed hypertensive during the initial survey (492), was chosen by simple random sampling.

#### Collection of data:

The field -assessment team comprised trained members from the Nicobarese community. An investigator, a medical doctor accompanied the team throughout the survey. A periodic cross-checking of the collected data was carried out by other investigator who was not in the survey team. Piloting and standardisation of the interview schedule was carried out before starting the survey.

#### 1. History:

(a) Hypertension awareness and treatment - With regard to hypertension awareness and treatment four questions were asked: (1) Whether the person’s blood pressure (BP) had ever been checked priorly; (2) Whether they were hypertensive; (3) whether they were told by a doctor of their hypertension status and (4) whether they were on regular (for more than past three months) treatment for hypertension. These questions were asked by a clinician.

(b) Structured interview schedule to capture the hypertension risk - The subjects were administered a pre-tested and structured interview schedule, reliably translated into Hindi (using standard translation-back-translation methods). It consisted of the following details:

Family proforma - A family proforma collected composite data about the family, from a key-informant (any adult member belonging to the family). All the individuals aged 18 and above were administered individual proforma.

Individual proforma - An individual proforma collected data pertaining to socio-demographic details of the individual like sex, age, and educational status. Tobacco consumption frequency and pattern was captured by using both smoking and smokeless tobacco use versions of Fagerstrom Nicotine Tolerance Questionnaire^17^,^18^. From the consumers of alcoholic beverages, the quantity of alcohol drunk on typical drinking occasions and the frequency of typical drinking were collected. The strength of alcohol in locally brewed beverages was measured and it was translated into local measurements (12 g of absolute alcohol = one standard drink). From this, Quantity × Frequency (QF) value for annual consumption of alcohol for individual consumer was calculated. The Alcohol Use Disorder Identification Test (AUDIT) was administered to all of them^19^.

#### 2. Clinical examination and examinations:

Blood pressure was measured by a clinician using sphygmomanometer. Three readings were taken over five minutes and lowest one was recorded. In the sitting position, the right hand was used consistently^20^. Every day before starting the survey the correctness of instrument was checked by measuring the BP of the six non-hypertensive field staff of the team. It was cross-checked by one more instrument kept as reserve. The same instrument was used throughout the survey. Wherever indicated (as per JNC 7 guideline^21^) anti-hypertensive medications were started with advice regarding compliance and follow up with local health services. For all the participants two things were advised irrespective of their hypertension status. Those were, to decrease salt consumption and to increase physical activity.

Weight was measured by lever activated electronic weighing machine with an accuracy of 100 g. Height was measured by anthropometry rod with an accuracy of 2 mm. The weighing machine was checked with known weights each day before starting the survey.

#### 3. Selection bias and generalisability:

To ascertain the generalisability of the study to the Nicobarese population living in Car Nicobar Island and to identify the selection bias, the proportion of study subjects in each sex category and different age category was compared with the census figure of 2001 for Car Nicobar Island, India^15^. The coverage rate attained in each tuhet and the broad demographic characteristics of those who have been not studied was also analysed. The blood pressure of 100 randomly selected subjects from those not studied was checked.

#### 4. Repeat survey:

During the repeat survey of the subpopulation of previously diagnosed hypertensive persons, in 2009, along with checking of BP, two questions were asked: (1) Are you taking medication as advised? and (2) if not, why not?. These were done by a clinician.

#### Diagnostic criteria for various risk factors and hypertension

Tobacco consumption and the Fagerstrom Nicotine Tolerance Questionnaire - This is a set of 6 questions having a maximum score of 10 which categorises the consumers of tobacco into various grades of dependence (0-3 = Low; 4-6= Medium; 7-10= High)^17^,^18^.

Alcohol consumption and the AUDIT - The AUDIT categorises consumers of alcohol into four different risk levels [0-7 = Low-risk; 8-15 = Risky or hazardous level; 16-19 = High risk or harmful level; 20 or more = High - risk)^19^.

Nutritional status (Body mass index) - The adults were classified into different grades of nutritional status as per the international classification of adult underweight, overweight, and obesity according to BMI proposed by WHO^22^,^23^. (BMI < 18.50 kg/m^2^ = underweight; BMI 18.50 to 24.99 = normal; BMI ≥ 25.0 to 29.99 = overweight; BMI ≥ 30 = obese).

Hypertension - Individuals with systolic blood pressure (SBP) of ≥ 140 and or diastolic pressure (DBP) of ≥ 90 and those who were already under medication were considered as hypertensive^23^.

#### Data analysis:

The data were analysed using the Statistical Package for Social Sciences (SPSS) soft ware (Version 17.0) (SPSS, Inc., Chicago, IL). The overall prevalence of hypertension in relation to various other variables along with 95 per cent confidence interval was calculated taking into consideration the two stage design of sampling. Bivariate analysis was carried out to find out associations between age, educational status, tobacco consumption, alcohol consumption, Fagerstrom score category, QF value category, AUDIT score category, nutritional status with hypertension. Odds ratio was calculated with 95 per cent confidence interval for the above variables. The increasing trend in the prevalence of hypertension with increasing age and decreasing educational status was analysed by χ^2^ for linear trend by using epi-Info version 3.4.3. (Centres for Disease Control and Prevention, Atlanta, USA). Multiple logistic regressions (MLR) analysis was carried out to examine the associations between all independent variables (age, education, alcohol consumption status, and nutritional status) with hypertension. The independent variables were entered into the equation all together.

## Results

### 

#### Prevalence of hypertension and association with risk factors:

Of the 975 subjects assessed, 492 (50.5%) (95%CI 46.1-54.9) were classified as hypertensive. Of the latter, 364 had a SBP of ≥ 140 mm Hg and a DBP of ≥ 90 mm Hg, 84 had only a SBP of ≥ 140 mm Hg, 43 had only a DBP of ≥ 90 mm Hg and 1 had BP under control with treatment. The prevalence of hypertension with 95 per cent CI, under various variable categories is depicted in Table I. The association of hypertension with various variables in the equation is shown in Table II. The association between hypertension and various age and education subcategories, alcohol consumption status, and overweight/obesity was statistically significant (*P* < 0.05). The increasing trend in the prevalence of hypertension with increasing age (χ^2^ for linear trend=95.88, *P*< 0.001) and decreasing educational status (χ^2^ for linear trend=25.55, *P*< 0.001) was statistically significant. Multiple logistic regression analysis revealed a significant association between hypertension and various age categories and overweight/obesity (*P* < 0.05) Table III.

**Table I:** Prevalence of hypertension among Nicobarese population

Variable (n)	Subcategory, n (%)	Hypertensive, n (%) [95% CI]

(975)		492, (50.5) † [46.1-54.9]
Sex (975)	Male, 424 (43.5)	215, (50.7) [44.0-57.4]
	Female, 551(56.5)	277, (50.3) [44.4-56.2]
Age (975) (yr)	18-29<,270 (27.7)	78, (28.9) [18.8-38.9]
	30-39<,251 (25.7)	109, (43.4) [34.1-52.7]
	40-49<,166 (17.0)	100, (60.2) [50.6-69.8]
	50-59<,152 (15.6)	105, (69.1) [60.3-77.9]
	≥60,136 (13.9)	100, (73.5) [64.8-82.2]
Education (824)	No-education,244 (29.6)	146, (59.8) [51.8-67.8]
	1-5 yr, 139 (16.9)	77, (55.4) [44.3-66.5]
	6-10 yr, 357 (43.3)	160, (44.8) [37.1-52.5]
	College,84 (10.2)	27, (32.1) [14.5-49.7]
Tobacco consumption (831)	No,100 (12.0)	48, (48.0) [33.9-62.1]
	Yes++,731 (88.0)	363, (49.7) [44.6-54.8]
Alcohol consumption (831)	No, 371 (44.6)	168, (45.3) [37.8-52.8]
	Yes,460 (55.4)	243, (52.8) [46.5-59.1]
KF score category (717)	≤3,483 (67.4)	234, (48.4) [42.0-54.8]
	4 – 6,210 (29.3)	111, (52.90 [43.6-62.2]
	≥7,24 (3.3)	11, (45.8) [16.4-75.2]
QF value category (458)	≤72 (50^th^ percentile),241 (52.6)	127, (52.7) [44.0-61.4]
	>72 (> 50^th^ percentile), 217 (47.4)	113, (52.1) [42.9-61.3]
AUDIT score category (451)	<8,347 (76.9)	185, (53.3) [46.1-60.5]
	8 – 15, 84 (18.6)	45, (53.6) [39.0-68.2]
	16 – 19, 10 (2.2)	5, (50.0) [6.2-93.8]
	≥20, 10 (2.2)	3, (30.0) [-]
Nutritional status (667)	Thinness, 34 (5.0)	11, (32.4) [4.7-60.0]
	Normal, 387 (58.0)	159, (41.1) [30.5-48.7]
	Overweight, 172 (25.8)	102, (59.3) [49.8-68.8]
	Obese, 74 (11.0)	62, (83.3) [74.0-92.6]

† The prevalence of hypertension adjusted to 2001 census was 47.2 per cent (Male: 46.4%; Female: 48.2%)

++ Chewing (smokeless tobacco)-81 per cent; smoking-6.7 per cent; both-12.3 per cent, KF, Karl Fagerstrom nicotine tolerance questionnaire score; QF, Quantity Frequency value; AUDIT, Alcohol Use Disorder Identification Test

**Table II:** Association of hypertension with various variables in the equation †

Variable	Bivariate analysis		
	Odds Ratio (OR)	95% Confidence Interval (CI)	*P* value

Age (yr)*			
18- 30 (Reference)	1.0		
≥60	6.83	4.30-10.86	<0.001
50-59	5.49	3.56-8.48	<0.001
40-49	3.73	2.48-5.60	<0.001
30-39	1.88	1.31-2.71	=0.001
Education** College (Ref.)	1.0		
Illiterate	3.14	1.86-5.31	<0.001
1-5 yr	2.62	1.48-4.62	=0.001
6-10 yr	1.71	1.03-2.83	=0.03
*Alcohol* No (Ref.)	1.0		
Yes	1.35	1.02-1.78	=0.03
*Body mass index (BMI)*			
Normal (Ref.)	1.0		
Overweight/obese	2.86	2.05-4.0	<0.001
Thinness	0.68	0.32-1.44	=0.32

† Similarly tobacco consumption status, KF score category, QF value category, and AUDIT score category were subjected to bivariate regression analysis but none were significant

* *χ*^2^ for linear trend=95.88, *P*< 0.001

** *χ*^2^ for linear trend=25.55, *P*< 0.001

**Table III:** Multiple Logistic Regression (MLR)*analysis

Variable	MLR analysis		
	Odds Ratio (OR)	95% Confidence Interval (CI)	*P* value

*Age*			
18- 30 (Reference)	1.0		
≥60	8.74	4.56-16.73	<0.001
50-59	8.17	4.37-15.29	<0.001
40-49	4.39	2.43-7.93	<0.001
30-39	1.66	1.00-2.75	=0.04
*Body mass index (BMI)*			
Normal (Ref.)	1.0		
Overweight/obese	4.10	2.72-6.19	<0.001
Thinness	0.29	0.11-0.79	=0.01

* Other variables included in the model were education and alcohol but not became significant

#### Hypertension awareness and treatment:

Four hundred and ninety two of 975 tested had hypertension (50.5%). Sixty of 492 (12.19%) were aware of their hypertension status due to a medical diagnosis by a doctor. Only 11 of 492 (0.02%) detected to be hypertensive were on regular treatment for hypertension (for more than past three months). However, the control was achieved in one person only.

The blood pressure in 340 of 975 (35%) subjects was checked for the first time in their lives. Three hundred and eleven of 975 (32%) subjects perceived themselves to be hypertensive. However, many, [251 of 975 (26%)] assumed themselves to be hypertensive based on the symptoms experienced by them like headache, giddiness, tiredness, and excessive sweating. Of the 311 subjects who perceived themselves to be hypertensive, 180 (58%) were found to be hypertensive.

#### Selection bias and generalisability:

The sex ratio of study subjects was 100: 130 (M: F) against the census figure (2001, Car Nicobar Island) of 100: 91 (M: F). Those aged ≥18-40 yr constituted 53.4 per cent (against census figure of 59.5%) and ≥ 40 yr constituted 46.4 per cent (against census figure of 40.5%).The prevalence of hypertension adjusted to 2001 census was 47.2 per cent (Male: 46.4%; Female: 48.2%). The coverage was > 75 per cent in 25 tuhets, and the overall coverage for the 40 tuhets was 77 per cent. The sex ratio of the remaining 295 subjects who were not covered was 100: 67 (M: F). Of these, 191 (64.7%) were aged ≥18-40 yr and 104 (35.3%) were ≥ 40 yr. Among 295 subjects, 85 were government employees posted outside Car Nicobar Island. Of the remaining 210 subjects, 100 were randomly chosen and their blood pressure was checked, 47 were found to be hypertensive (47%).

#### The repeat survey:

During the repeat survey 248 of 250 (99%) had hypertension (245 had systolic blood pressure of ≥140 and or diastolic pressure ≥ 90 mm of Hg and 3 were on medication). All were aware of their hypertension status. Only 3 of 248 (0.01%) subjects were under regular treatment and in all of them the hypertension was under control. Almost every body (247 of 250) stopped medication because they thought it might have got cured now as they took the medication for more than two weeks when it was initially prescribed. Also, 150 of 250 (60%) subjects stopped medication because there were no symptoms which hamper their day to day activity in addition to above mentioned reason.

## Discussion

The present study documents a high prevalence of hypertension (50.5%) and its determinants like overweight/obesity (37%), tobacco (88%) and alcohol consumption (54%), and illiteracy (30%) among the Nicobarese population. The study also reveals poor awareness (12%), treatment (0.01%), and control (1 of 492) with extremely poor adherence to therapies (0.01%).

Though the present study has thrown light on the hitherto unknown health problem of the community, it is of preliminary nature with limitations. The lifestyle risk factors like diet, dietary salt, fibre, saturated fat, trans-fat, physical activity or stress among the Nicobarese community were not estimated. It is well known that factors like dietary salt consumption can influence the BP independent of other risk factors^24^. Also, though it was the target of the study to get the data of risk factors from all 975 subjects whose BP was measured but it could not be achieved because of various socio-cultural beliefs of the community. All the adult members available in the tuhet were investigated but those who had gone to forest, fishing or out of the island (predominantly males) for employment opportunities were missed. This is reflected in over representation of older age group and female subjects in the study. Also, there is slight under-representation of younger age group. But, the fact that prevalence of hypertension adjusted to 2001 census was 47.2 per cent and 47 of 100 those who have been not studied tested hypertensive proves that findings of present study was not affected by these limitations. Our aim was to choose an appropriate sample size for an expected prevalence of 25 per cent with an absolute precision of 5 per cent, and this was achieved as the reported estimate was 50.5 per cent with an absolute precision of 4.4 per cent. Also, the post-facto computed design effect was 1.6 against the assumed design effect of 3.5. Since almost 90 per cent Nicobarese tribes people live in this island, this study has a generalisability value with regard to Nicobarese tribes’ people living in Car Nicobar Island. An other limitations was that the diagnosis was made only on one reading of blood pressure and the incomplete follow up data.

It is increasingly been recognized that the poor, marginalized, and tribal communities are facing the burden of non-communicable diseases in general and hypertension in particular in India^5^,^10^–^13^. Studies carried out among the Lepchas of Sikkim Himalayas has documented hypertension prevalence of 30.77 per cent among males and 25.77 per cent among females (By using older WHO criteria for hypertension)^10^. The most recent composite national data of NNMB has documented hypertension prevalence of 25 per cent among rural adults^5^. In the present study the prevalence of hypertension among Nicobarese was high and was much more than the rural and urban populations in India. The prevalence documented in the present study is almost double than that in many African population groups^9^,^25^. Though the usage of tobacco and alcohol was very high among Nicobarese but it did not add to the risk of hypertension. It has to be noted that majority consumed smokeless tobacco (almost 94% among consumers). The association between smokeless tobacco use and risk of hypertension lacks evidence^26^,^27^. Similarly, majority of the consumers of alcohol (77%) fitted into a low risk category (AUDIT score < 8). The prevalence of overweight/obesity was also high among Nicobarese compared to rest of India^5^. The rapid nutrition survey carried out by Hyderabad, India in March 2005 among tsunami affected population living in the relief camps in Andaman and Nicobar Islands has revealed high prevalence (25%) of overweight/obesity among Nicobarese^28^. The present study further substantiates the previous finding. The awareness of hypertension (medically diagnosed) status was comparable to the studies done almost a decade back in rural India^29^.

In conclusion, the present findings show high prevalence of hypertension among Nicobarese tribal population. Nutritional transition and gene - environment interaction is blamed for high prevalence of hypertension in the recent times among marginalised, poor, and tribal communities. Among Nicobarese this needs research to decipher the exact dynamics.
